# Euglena extract suppresses adipocyte-differentiation in human adipose-derived stem cells

**DOI:** 10.1371/journal.pone.0192404

**Published:** 2018-02-15

**Authors:** Ryota Sugimoto, Naoko Ishibashi-Ohgo, Kohei Atsuji, Yuko Miwa, Osamu Iwata, Ayaka Nakashima, Kengo Suzuki

**Affiliations:** Department of Research and Development, euglena Co., Ltd., Minato-ku, Tokyo, Japan; INIA, SPAIN

## Abstract

*Euglena gracilis Z* (Euglena) is a unicellular, photosynthesizing, microscopic green alga. It contains several nutrients such as vitamins, minerals, and unsaturated fatty acids. In this study, to verify the potential role of Euglena consumption on human health and obesity, we evaluated the effect of Euglena on human adipose-derived stem cells. We prepared a Euglena extract and evaluated its effect on cell growth and lipid accumulation, and found that cell growth was promoted by the addition of the Euglena extract. Interestingly, intracellular lipid accumulation was inhibited in a concentration-dependent manner. Quantitative real-time PCR analysis and western blotting analysis indicated that the Euglena extract suppressed adipocyte differentiation by inhibiting the gene expression of the master regulators peroxisome proliferator-activated receptor-γ (PPARγ) and one of three CCAAT-enhancer-binding proteins (C/EBPα). Further Oil Red O staining experiments indicated that the Euglena extract inhibited the early stage of adipocyte-differentiation. Consistent with these results, we observed that down-regulation of gene expression was involved in the early stage of adipogenesis represented by the sterol regulatory element binding protein 1 c (SREBP1c), two of three CCAAT-enhancer-binding proteins (C/EBPβ, C/EBPδ), and the cAMP regulatory element-binding protein (CREB). Taken together, these data suggest that Euglena extract is a promising candidate for the development of a new therapeutic treatment for obesity.

## Introduction

Obesity is an abnormal health condition in which body fat is excessively accumulated [1, 2]. Various recent studies have reported that obesity is associated with several chronic diseases, such as type 2 diabetes mellitus (T2DM), asthma, and cardiovascular disease [3–5]. Strikingly, it was reported that there was a positive correlation between body mass index and mortality caused by those diseases [6, 7]. Contrary to these aspects, adipocytes have crucial functions that contribute to the maintenance of lipid homeostasis [8] and intracellular energy balance by storing/releasing triglycerides or fatty acids, depending on the extracellular environment [9, 10]. Thus, to understand the physiology of adipocytes, many studies involving adipocyte-differentiation and its regulatory system have been carried out in the murine 3T3-L1 cells and *in vivo*.

The peroxisome proliferator-activated receptor-γ (PPARγ) and the CCAAT-enhancer-binding protein family (C/EBPα, C/EBPβ, C/EBPδ), particularly C/EBPα, have been identified as master regulators that control adipocyte-differentiation, and thus, their physiological roles have been widely analyzed [11]. When cells are exposed to isobutylmethylxanthine (IBMX) or dexamethasone (DEX), *C/EBPβ* and *C/EBPδ* are expressed immediately, resulting in the upregulation of *PPARγ* [12, 13]. Activated PPARγ induces the expression of *C/EBPα*, which turns on several genes involved in adipocyte-differentiation to mature adipocyte [14].

In addition to C/EBPβ and C/EBPδ, several studies have revealed the physiological role of the sterol regulatory element binding protein 1 (SREBP1), which is associated with adipogenesis, insulin sensitivity, and fatty acid metabolism [15]. SREBP1 exists as two isozymes (SREBP1a and SREBP1c) derived from alternative splicing of the first exon [16]. SREBP1a is predominantly expressed in the spleen, whereas SREBP1c is predominantly expressed in the adipose tissue, liver, adrenal glands, and skeletal muscle in response to insulin [17]. *In vitro* experiments suggested that SREBP1c contributes to adipocyte-differentiation through the activation of *PPARγ* as well as C/EBPβ and C/EBPδ, either through the induction of enzymes responsible for endogenous ligand generation and/or by enhancing gene expression [18, 19]. Not only C/EBPβ and C/EBPδ, but also the cAMP regulatory element-binding protein, CREB, which also participates in the early stage of adipocyte-differentiation. Klemm and Lane reported that CREB is activated early during adipocyte-differentiation in response to isobutylmethylxanthine, which increases cellular cAMP in 3T3-L1 cells, resulting in the activation of C/EBPβ [20, 21].

PPARγ is a functional receptor for insulin-sensitizing drugs known as thiazolidinediones, which are used for the treatment of T2DM [22]. Anti-obesity drugs such as sibutramine and orlistat were developed after much pharmacological research to prevent and reduce obesity. However, it has been indicated that intake of these drugs might cause serious adverse reactions such as insomnia, constipation, headache, and cardiovascular stroke [23, 24]. Thus, many researchers have sought anti-obesity drugs that do not cause such adverse reactions. For instance, it has been reported that coffee or seaweed extract suppresses the gene expression of PPARγ and C/EBPα as well as lipid accumulation in the 3T3-L1 cell line, but reductions in cell viability were observed when these additives were used at high concentrations [25, 26]. However, studies on adipocyte -differentiation in human-derived stem cells have been limited. Thus, the development of anti-obesity drugs without adverse reactions against human-derived cells would be promising.

*Euglena gracilis Z* (Euglena) is a unicellular, photosynthesizing, microscopic green alga found in fresh water. Several studies revealed that the alga belongs to the phylum Euglenozoa, which is a member of Excavata, a root of eukaryote [27, 28]. Furthermore, it is also known that Euglena contains several nutrients such as vitamins, minerals, and unsaturated fatty acids. Moreover, it accumulates the reserve polysaccharide clystalline β-1,3-glucan, known as paramylon, which is considered a functional dietary fiber for health purposes [29, 30]. Therefore, the effect of consumption of Euglena and/or paramylon on human health has been assessed in various studies. For example, it was reported that Euglena consumption alleviated hyperglycemia in OLETF rats [31] and that paramylon reduced the development of atopic dermatitis-like skin lesions in NC/Nga mice [32]. However, to our knowledge, there has been no report on the effect of Euglena consumption on obesity in human-derived stem cells. In this study, as part of finding a new effect of Euglena on human cell, the effects of Euglena on adipocyte-differentiation and lipid accumulation in human adipose-derived stem cells (hASCs) were investigated.

## Materials and methods

### Preparation of Euglena extract

Euglena dry powder was obtained from euglena Co., Ltd. (Tokyo, Japan), and 0.5 g of powder was suspended in 20 mL of water and heated at 95°C for 2 h. For removal of the insoluble fraction and sterilization, the supernatant was collected after centrifugation and was filtered with a 0.45-μm filter purchased from Merck Millipore Co., Ltd. (Billerica, MA, USA). The sterilized supernatant (called Euglena extract) was used as 100% of concentration and diluted by any concentrations with medium before cultivation of cells. The Euglena extract was stored at 4°C until use.

### Cell culture and differentiation

hASCs were purchased from Lonza Walkersville, Inc. (Walkersville, MD, USA); the cells were derived from a female donor (non-diabetic, BMI 26 kg/m^2^, 36 years old) (PT5006 lot 0000410257). The cells (passage 4) were pre-cultured as described by Yamada et al. [33]. Briefly, 2 × 10^4^ cells/mL of the cells were cultured in 50% Dulbecco’s modified Eagle’s medium (DMEM)/50% α minimum essential medium supplemented with 1% fetal bovine serum (FBS), 1 × ITS, 10 ng mL^-1^ bFGF (PeproTech Inc., NJ, USA), and 400 ng mL^-1^ of hydrocortisone on a 24-well plate for 3 days, until cells became confluent. To induce adipocyte-differentiation, post-confluent preadipocytes were stimulated in MDI differentiation medium (DMEM containing 10% FBS, 1 μM DEX, 0.5 mM IBMX, 0.2 mM indomethacin, 10 μg mL^-1^ insulin, and 33 μM biotin) for 7 days (Day 0–7). During cultivation, the culture was replaced with fresh MDI differentiation medium every 2 days with or without Euglena extract. Then, cells were maintained for a further 7 days (Day 7–14) in adipocyte nutrition medium (DMEM containing 10% FBS, 10 μg mL^-1^ insulin, and 33 μM biotin), which was replaced with fresh culture every 2 days. All cells were cultured at 37°C under a 5% CO_2_ atmosphere. All the above reagents were purchased from Sigma-Aldrich (St. Louis, MO, USA).

### Determination of cell viability

The cytotoxicity of Euglena extract against hASCs was estimated by a modified MTT assay using the Cell Counting-kit 8 (Dojindo, Kumamoto, Japan); 100% confluent hASCs preadipocytes were cultured in D/α (-) medium (50% DMEM/50% α minimum essential medium (αMEM) supplemented with 1% FBS, 1 × ITS, and 400 ng mL^-1^ hydrocortisone) on a 96-well plate were treated with various concentrations of water or Euglena extract (1.25%, 2.5%, 5%, 10%, 20%, 40%) at 37°C for 48 h under a 5% CO_2_ atmosphere. 100 μL of the Cell Counting-Kit 8 solution was added to cells after aspirating the culture supernatant. After incubation for 30 min at 37°C under a 5% CO_2_ atmosphere, absorbance was measured at 450 nm with the microplate reader SH-1200Lab (Hitachi High-Tech Science Co., Ltd., Tokyo, Japan). Cell viability was determined by comparison of absorbance with the absorbance of the cells treated with no additives as control.

### Estimation of lipid accumulation by staining with Oil Red O solution

After adipocyte-differentiation in MDI differentiation medium and adipocyte nutrition medium without or with various concentration of water (5%, 10% or 20%) or Euglena extract (5%, 10% or 20%) for 14 days (Day 0–14) or 7 days (Day 0–7 or Day 8–14) dependent on assay estimating the effect of the extract on lipid accumulation at early or late stage of adipocyte-differentiation. The cells fixed with 4% paraformaldehyde were washed with 500 μL phosphate-buffered saline (PBS), and 500 μL of 60% isopropanol was added to each well. After aspirating the culture supernatant, cells were treated with Oil Red O solution (0.3 g in 100 mL isopropanol; Sigma-Aldrich) for 15 min at room temperature, and the cells were photographed. To estimate adipogenesis, after removing the staining solution, the dye retained in the cells was eluted into 500 μL isopropanol, and absorbance was measured at 520 nm. Oil Red O content of cells was calculated from comparison with the absorbance of the cells treated with no additives as control for induction of adipocyte-differentiation.

### RNA extraction and analysis of gene expression

Total RNA was extracted from cells by RNAiso plus (TaKaRa, Shiga, Japan) according to the manufacturer’s instructions. cDNA was synthesized from 200 ng of total RNA using PrimeScript Master Mix (TaKaRa) PCR primers are listed in Table 1 and S1 Table. Quantitative real-time PCR (RT-qPCR) was performed in StepOne Plus using the PowerUp SYBR Green Master Mix (Applied Biosystems Inc., Warrington, UK). The amount of target gene relative to the reference gene (*GAPDH*) was calculated based on the comparative threshold (Ct) method [34]. All data were normalized with the amount of GAPDH at induction of adipocyte-differentiation (Day 0).

**Table 1:** Primers used in quantitative RT-qPCR.

Primers		Sequence (direction: 5´ to 3´)
PPARγ	Forward	GAACGACCAAGTAACTCTCCTCAAAT
	Reverse	TCTTTATTCATCAAGGAGGCCAGCATT
C/EBPα	Forward	GGGTCTGAGACTCCCTTTCCTT
	Reverse	CTCATTGGTCCCCCAGGAT
C/EBPβ	Forward	GACAAGCACAGCGACGAGTA
	Reverse	AGCTGCTCCACCTTCTTCTG
C/EBPδ	Forward	TTCAGCGCCTACATCGACTC
	Reverse	TTGAAGAGGTCGGCGAAGAG
SREBP1c	Forward	TTGAGGACAGCAAGGCAAAG
	Reverse	GGACAGGCAGAGGAAGACGA
CREB	Forward	ACGAAAGCAGTGACGGAGGA
	Reverse	TACGGTGGGAGCAGATGATG

### Western blotting analysis

Western blotting analysis was conducted to identify the gene expression of adipogenesis master regulator proteins, PPARγ and C/EBPα. Preparation of cell lysate and western blotting analysis were conducted as described by Aji et al. [35]. Briefly, cells were lysed in RIPA buffer (Wako) containing cOmplete EDTA-free Protease Inhibitor Cocktail (Roche, Mannheim, Germany) for 15 min on ice before sonication. The obtained cell lysate was centrifuged at 12,000 rpm for 15 min at 4°C, and the supernatant was sampled. The protein concentration was evaluated by using the BCA method (TaKaRa). Approximately 25 μg of protein samples were loaded on a 12% SDS-PAGE after denaturation at 95°C for 5 min. After electrophoresis, the separated proteins were transferred to a polyvinylidene difluoride membrane by using WSE-4115 PoweredBlot Ace (ATTO Co. Ltd., Tokyo, Japan), and the membrane was blocked by incubation in TBS-(T) (TBS containing 0.1% Tween 20 (Sigma-Aldrich)) and 5% skimmed milk for 1 h. The membrane was probed with antibodies against PPARγ (1:1000, Cell Signaling TECHNOLOGY, MA, USA), C/EBPα (1:1000, Abcam, Cambridge, UK), or GAPDH (1:2500, Abcam) as an internal loading control overnight at 4°C. The membrane was washed three times with TBS-(T) before treatment with antibody against rabbit IgG conjugated with horseradish peroxidase (1:5000, Abcam) for 1 h at room temperature. Signals were developed using an Amersham ECL western blotting analysis system (GE Healthcare, Little Chalfont, UK) by using MyECL imager (Thermo Fisher Scientific, MA, USA).

### Statistical analysis

All data are represented as mean ± SEM from 3 independent experiments. Statistical significance was analyzed using Student’s *t*-test, and *P* < 0.05 was considered significant.

## Results

### Euglena extract inhibits lipid accumulation in adipocyte-differentiation

**Fig 1:**
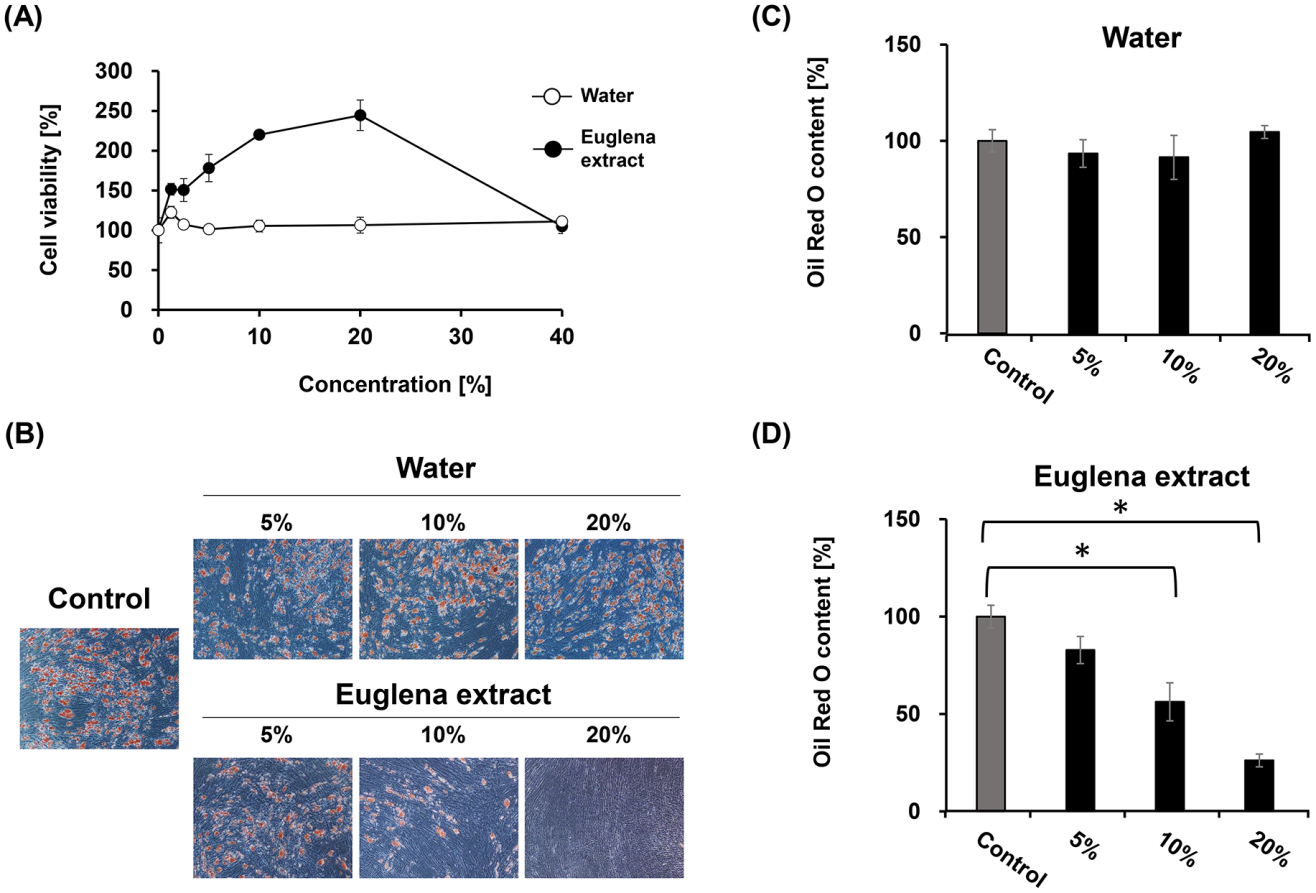
Euglena extract reduces lipid accumulation in hASCs. (A) Effect of Euglena extract on cell viability. The confluent hASCs were cultured in D/α (-) medium with water (white circles) or Euglena extract (black circles) for 2 days at 37°C under 5% CO_2_ atmosphere before WST-8 assay. (B) Microscopic images of hASCs stained with Oil Red O. (C) and (D) Relative abundance of accumulated Oil Red O in hASCs treated with water (C) or Euglena extract (D) compared with control (no additive, dark grey bar in (C) and (D)). Data represent the mean ± SEM from 3 independent experiments. *P <0.*05* (Student’s *t*-test).

To verify the effects of Euglena on cytotoxicity and lipid accumulation in hASCs, it was considered that dissolving Euglena dry powder into the culture would have been convenient for estimating its effects, but the dry powder could not be dissolved into the cultures. Thus, we prepared a Euglena extract by eluting the powder with water at 95°C. First, to evaluate the effect of the extract on cytotoxicity, hASCs (passage 4, 2 × 10^4^ cells/mL) were cultivated to confluence on DMEM for 3 days, and DMEM was replaced with D/α (-) medium, which maintains the cells. Simultaneously, various concentrations of Euglena extract (1.25%, 2.5%, 5%, 10%, 20%, 40%), water (1.25%, 2.5%, 5%, 10%, 20%, 40%), or without these materials as a control were added to the medium. After the cells were cultured for 48 h, a WST-8 assay was conducted to estimate viable cells and compared to control. Cytotoxicity was not observed by the addition of water or Euglena extract into the culture. Interestingly, treatment with Euglena extract increased cell viability in a concentration-dependent manner, even when cells were at confluence, which raised to approximately 250% compared to control at 20% of concentration (Fig 1(A)). It was reported that some adipose tissue-derived stem cells exhibited apparent lack of contact inhibition and piled up in extended culture [36]. Notably, the hASCs used in this study also exhibited this characteristic in the presence or absence of Euglena extract in D/α (-) medium. This result indicated that Euglena extract exhibited no cytotoxicity against hASCs.

Euglena extract may represent a source of nutrition for hASCs. To determine whether the Euglena extract inhibited lipid accumulation, confluent hASCs were stimulated with MDI differentiation medium to induce adipocyte-differentiation for 7 days (Day 0 to Day 7). Simultaneously, various concentrations of Euglena extract (5%, 10% or 20%), water (5%, 10% or 20%), or without these materials as control were added to the medium on Day 0. Stimulated cells at Day 14 were stained with Oil Red O and photographed (Fig 1(B)–1(D)). Compared with control, the cells treated with water contained Oil Red O as well as control, indicating that there was no effect on adipocyte differentiation (Fig 1(C)). Interestingly, the Oil Red O content was reduced to 83%, 56%, and 26% by the addition of Euglena extract at concentrations of 5%, 10%, and 20%, respectively (Fig 1(B) and 1(C)). In an attempt to identify the effective compound(s) in the Euglena extract, we prepared and tested another Euglena extract eluted with dimethyl sulfoxide (DMSO extract), and the DMSO extract was added into MDI differentiation medium at various concentrations (1.25%, 2.5%, 5%, or 20%). The cell viability significantly reduced at more than 2.5% of concentration of DMSO, but in the case of DMSO extract, cell piling was observed by 2.5% (S1(A) Fig). Intriguingly, DMSO extract had no effect on adipocyte-differentiation compared with only DMSO (S1(B) and S1(C) Fig). Furthermore, when using Euglena extracts eluted at various temperatures (25°C, 50°C, 75°C, or 120°C), the observed inhibition of adipocyte differentiation was reproduced in all extracts (S2 Fig).

### Euglena extract suppresses gene expression of master regulators of adipocyte-differentiation *PPARγ* and *C/EBPα*

**Fig 2:**
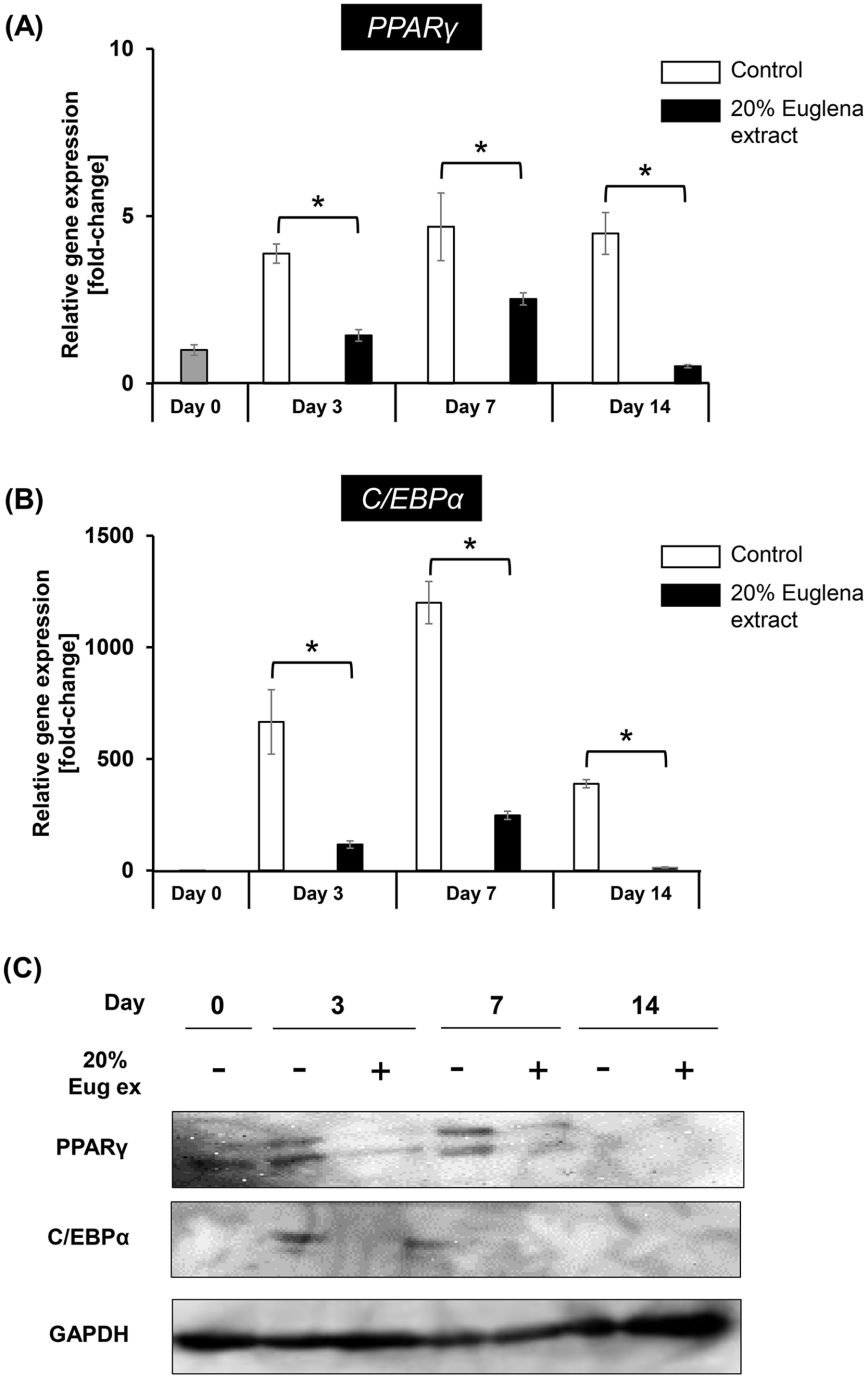
Euglena extract represses the gene expression of master regulators of adipocyte-differentiation. Relative gene abundances of (A) *PPARγ* and (B) *C/EBPα* in hASCs after treatment with no additive (control) or 20% Euglena extract for Day 3, Day 7, or Day 14 were monitored by RT-qPCR. Data represent mean ± SEM (n = 3). *P < 0.05 vs. control (Student’s *t*-test). (C) Western blotting analysis of PPARγ, C/EBPα, and GAPDH (as internal control). Cell lysate (25 μg) derived from soluble fraction was loaded in each lane. The data shown in (C) are representative from 3 independent experiments. Day 0 shown in (A)-(C) means before induction of adipocyte-differentiation.

Hitherto, it was indicated that treatment of hASCs with Euglena extract inhibited lipid accumulation by arresting adipocyte-differentiation. Based on this observation, it was speculated that the gene expression of master regulators involved in adipocyte-differentiation, especially PPARγ and C/EBPα, would be inhibited by Euglena extract. To verify the hypothesis, the relative mRNA abundances was measured by RT-qPCR. Cells were cultured in MDI differentiation medium with or without 20% Euglena extract from Day 0 to 7, and then, the MDI differentiation medium was replaced with adipocyte nutrition medium with or without 20% Euglena extract on which cells were cultured for a further 7 days (Day 8–14). During cell cultivation, MDI differentiation medium and adipocyte nutrition medium were exchanged with fresh medium with or without 20% Euglena extract every 2 days. cDNA was synthesized from mRNA extracted on Day 0, Day 3, Day 7, or Day 14 and was used for RT-qPCR. As a result, in controls, the gene expression levels of *PPARγ* and *C/EBPα* were elevated during adipocyte-differentiation. Conversely, the gene expression levels of *PPARγ* and *C/EBPα* were repressed by 23% on average (Fig 2(A) and 2(B)). Consistent with this result, the protein amount of PPARγ and C/EBPα was significantly reduced by the addition of Euglena extract in adipocyte-differentiation (Fig 2(C)). It is reported that PPARγ and C/EBPα regulate the adipocyte-differentiation marker gene represented to aP2, adiponectin, and LPL, lipoprotein lipase, which promote lipid accumulation to form mature adipocytes [37]. Therefore, it was expected that the gene expression of aP2 and LPL was also reduced. Further RT-qPCR analysis supported this hypothesis, because treatment with Euglena extract on adipocyte-differentiation inhibited the gene expression of aP2 and LPL (S3 Fig). These results indicated that the inhibitory effect of Euglena extract on adipocyte-differentiation was occurring through suppression of master regulators.

**Fig 3:**
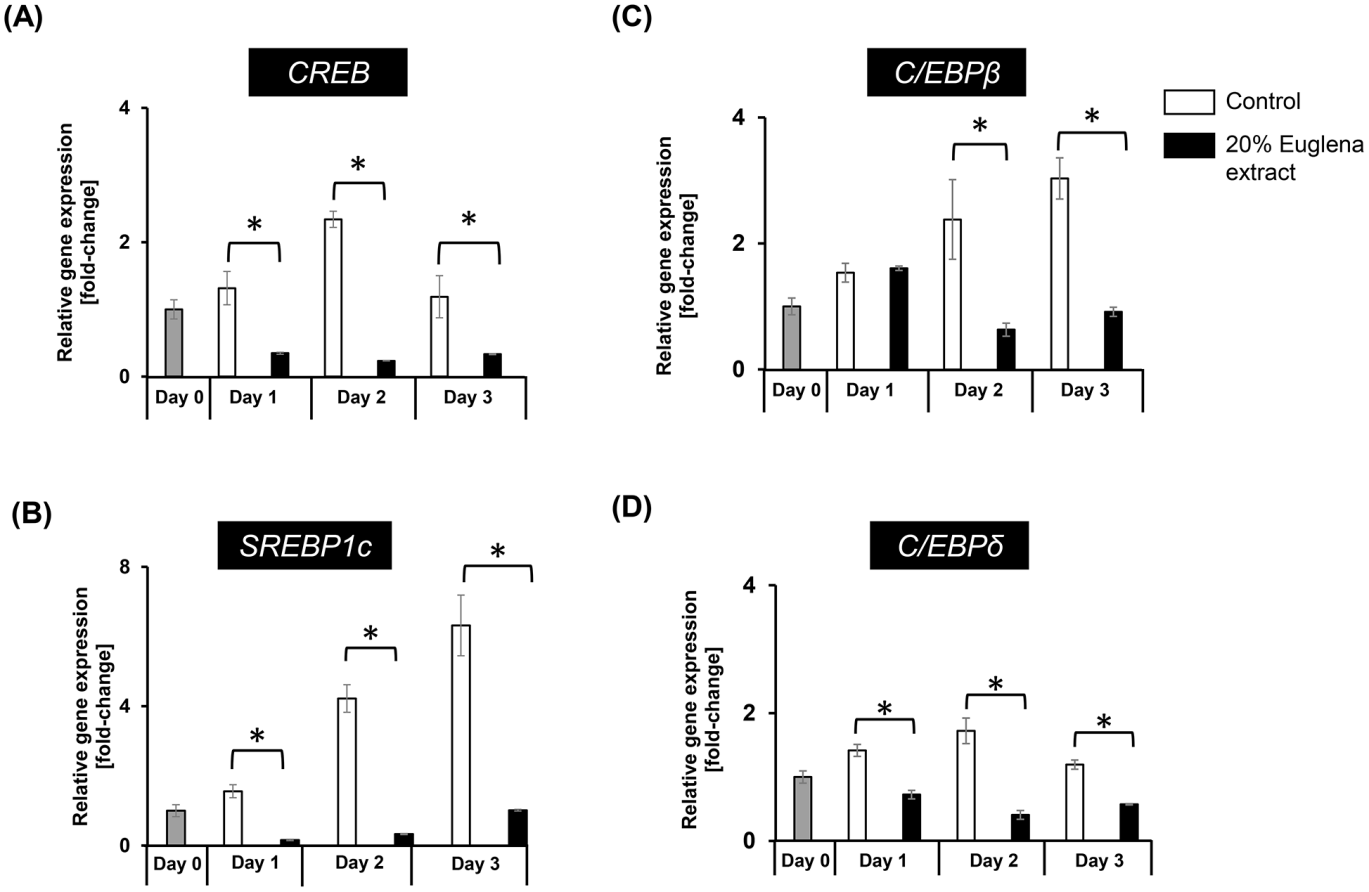
Expression of genes involved in the early stage of adipocyte-differentiation was suppressed by Euglena extract. Relative gene abundances of (A) CREB, (B) SREBP1c, (C) C/EBPβ, and (D) C/EBPδ in hASCs after treatment with no additive (control) or 20% Euglena extract for Day 3, Day 7, or Day 14 were monitored by RT-qPCR. Day 0 indicates before induction of adipocyte-differentiation. Data represent the mean ± SEM (n = 3). *P < 0.05 vs. control (Student’s *t*-test).

The expression of PPARγ is also activated by C/EBPβ and C/EBPδ at the early phase of adipocyte-differentiation [12, 13]. It is also known that SREBP1c and CREB are involved in the activation of PPARγ expression [18, 19]. To clarify the inhibitory effect of Euglena extract on early stage of adipocyte-differentiation, the abundance of mRNA of CREB, SREBP1c, C/EBPβ, and C/EBPδ was evaluated by using RT-qPCR, after cell cultivation for 1 day (Day 1), 2 days (Day 2), and 3 days (Day 3) with stimulation to differentiate adipogenesis. The gene expression level of CREB and SREBP1c was reduced by the addition of Euglena extract by 22% or 11% on average compared to control (Fig 3(A) and 3(B)). Not only CREB and SREBP1c were affected, but the gene expression level of C/EBPβ and C/EBPδ was also reduced by 48% and 50% on average, respectively. Taken together, these findings indicate that the inhibitory effect of Euglena extract on adipocyte-differentiation was caused by repressing the early stage of adipocyte-differentiation.

### Early stage of adipocyte-differentiation was inhibited by Euglena extract

**Fig 4:**
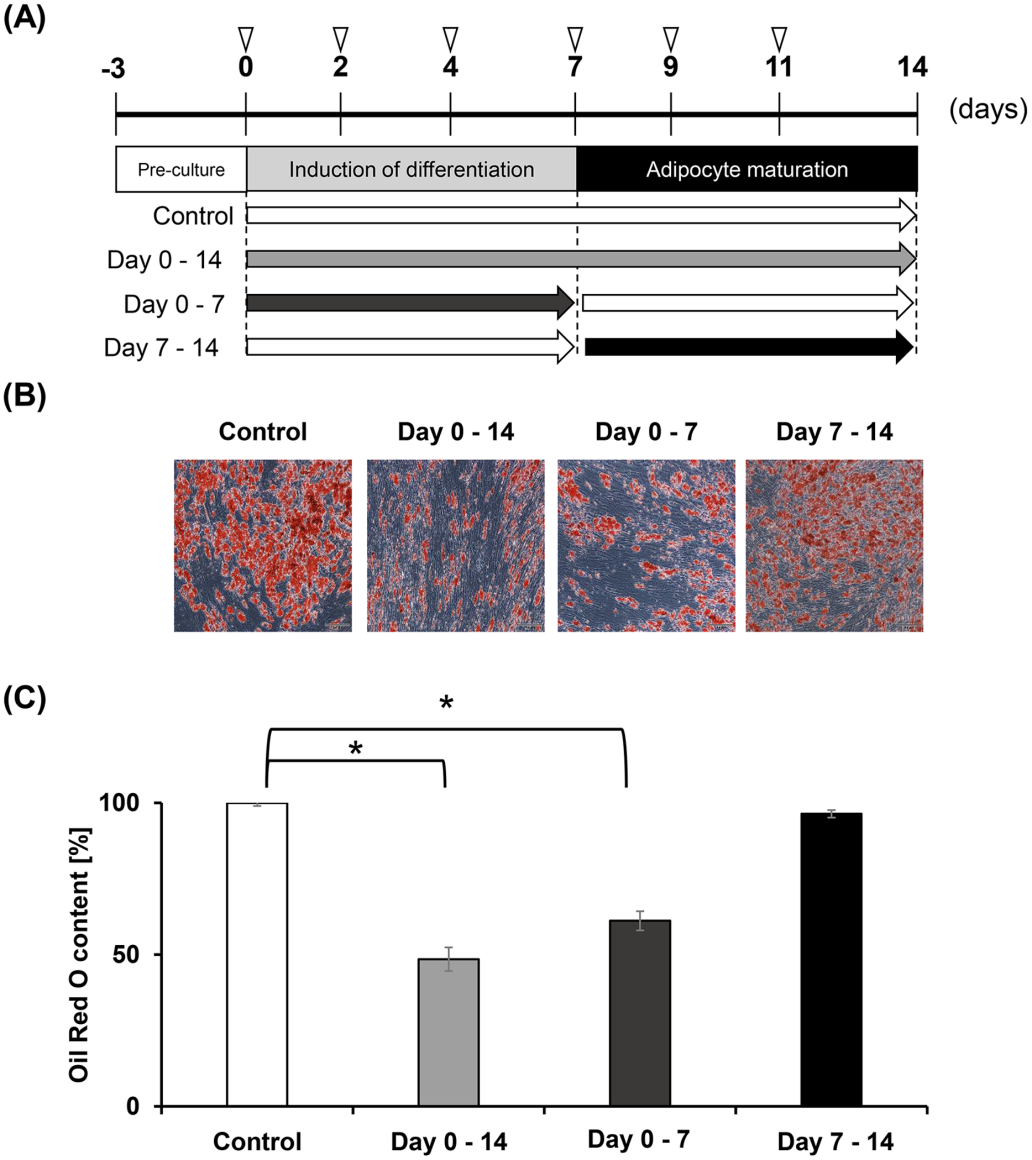
Euglena extract suppresses adipocyte-differentiation. (A) Experimental scheme. Inverted triangles show medium exchange points with or without Euglena extract. White bar shows no additive as control. Euglena extract (20%) was added to hASCs during adipocyte differentiation and maturation (Day 0–14, grey bar), induction of differentiation only (Day 0–7, dark grey bar), or adipocyte maturation only (Day 7–14, black bar). (B) Microscopic images of hASCs stained with Oil Red O. (C) Relative Oil Red O content in hASCs treated with Euglena extract compared with controls (no additive). Data represent mean ± SEM (n = 3). *P < 0.05 vs. control (Student’s *t*-test).

To verify the physiological role for the Euglena extract on the early stage of adipocyte-differentiation, adipogenesis was evaluated by eluting Oil Red O from cells treated with 20% of extract during adipocyte-differentiation and from those cells treated during adipocyte maturation (Fig 4(A)). It was speculated that if the extract had an inhibitory effect on adipocyte-differentiation, supplementation on Day 0–7 would be crucial for lipid accumulation, while if the extract exhibited the inhibition effect on adipogenesis, supplementation on Days 7–14 would be crucial. Compared with controls, constant supplementation (Day 0–14) with Euglena extract inhibited lipid accumulation by approximately 50%. Supplementation with extract on Day 0–7 also inhibited lipid accumulation by approximately 60%. Notably, approximately 96% of the accumulated lipids remained in the cells that were treated with the extract on Day 7–14 (Fig 4(B) and 4(C)). These results could support that Euglena extract suppresses adipocyte-differentiation at the early stage.

## Discussion

**Fig 5:**
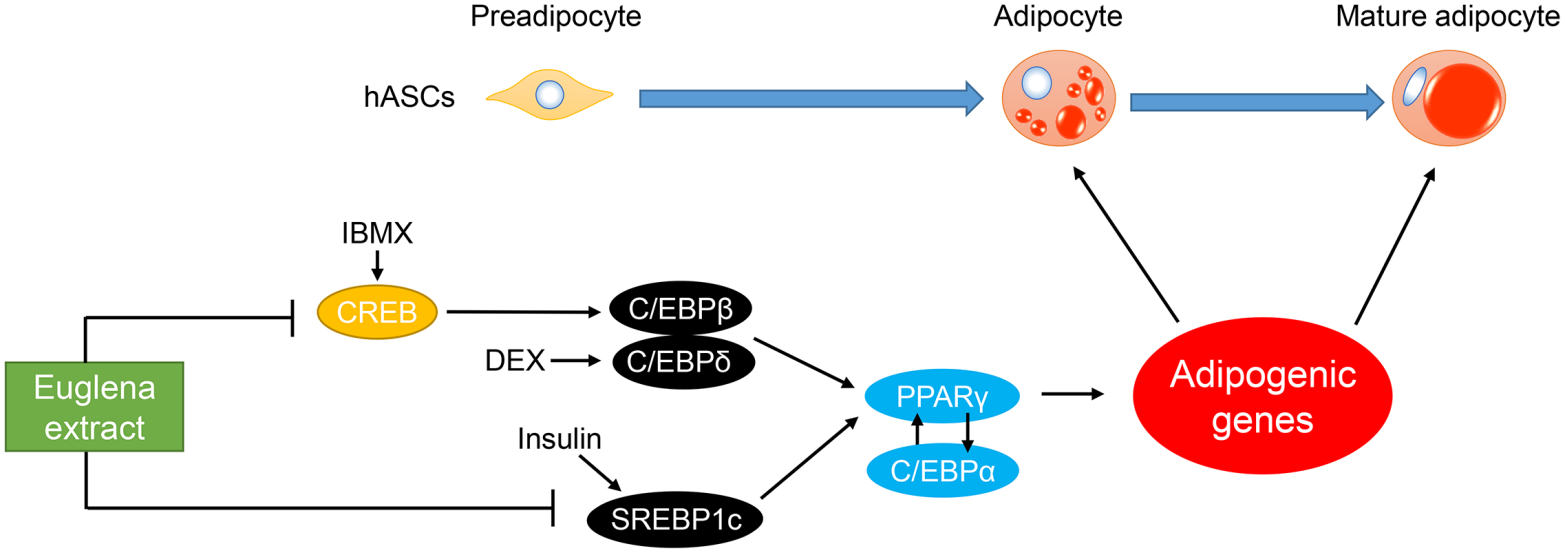
Proposed working model for the inhibitory effect of Euglena extract on adipocyte-differentiation. In the early phase of adipogenesis, CREB activated by IBMX induces the expression of C/EBPβ, and C/EBPδ is induced by DEX [12, 13, 20]. Another transcription factor, SREBP1c, is induced by insulin [17]. These transcription factors induce PPARγ, which also induces C/EBPα. Cross-regulation exists between PPARγ and C/EBPα and is considered a key component of transcriptional control for adipogenic genes [14]. Our observations indicate that Euglena extract inhibits the gene expression of *CREB* and *SREBP1c*, resulting in the downregulation of *PPARγ* and *C/EBPα* and inhibition of adipocyte-differentiation (Figs 2 and 3).

Algae have been increasingly recognized as resources of bioactive compounds for the improvement of human health [38]. Several recent studies have revealed that treatment with Euglena improves hyperglycemia and induces apoptosis of lung and breast cancer cells [31, 39]. However, to our knowledge, there has been no report showing the influence of Euglena on adipogenesis in hASCs. In the present study, the inhibitory effect of Euglena extract on adipocyte-differentiation in hASCs was evaluated, and the Euglena extract was capable of inhibiting adipocyte-differentiation without cytotoxicity (Fig 1). The regulatory mechanism of adipocyte-differentiation, in particular, the roles of the C/EBPα and PPARγ has been well documented [12, 40, 41]. RT-qPCR analysis and western blotting analysis for C/EBPα and PPARγ revealed that the Euglena extract inhibited these master regulators for adipocyte-differentiation at mRNA and protein levels, and further analysis indicated that other regulator genes of early stage of adipogenesis, C/EBPβ, C/EBPδ, and SREBP1c, were suppressed by the Euglena extract (Figs 2 and 3). Consistent with these results, it might be suggested that the Euglena extract inhibited lipid accumulation at the early stage of adipocyte-differentiation (Fig 4). Therefore, the above results allowed us to propose a working model for the effect of Euglena extract (Fig 5). However, the mechanisms underlying our observations remain unclear.

In the search for natural products having an antiobesity effect, several researchers have reported that coffee, extract from red and brown algae, pomegranate seed, or brown seaweed exhibited an antiobesity effect [25, 26, 42, 43]. Although fucoxanthin derived from brown algae and xanthigen, the mixture of fucoxanthin and punicic acid, derived from pomegranate seed and brown seaweed, have been known to significantly suppress adipocyte-differentiation through an inhibition of gene expression of the PPARγ and C/EBPs family [44, 45], there is no report that Euglena is capable of synthesis of these compounds. To identify the antiobesity compound(s) in the Euglena extract, a metabolome analysis was conducted, but fucoxanthin and punicic acid were not detected, with the exception of basic metabolites such as carbohydrates, amino acids, vitamins, and nucleic acids.

One possibility for the mechanism underlying the downregulation of *C/EBPβ*/*δ* by the Euglena extract could be that some compounds inhibited the enzymatic activity or gene expression of CREB, which participates in the induction of *C***/***EBPβ*/*δ* [46, 47]. Recently, lanostane, a triterpene derived from the lanosterol found in the fruiting bodies of *Ganoderma lucidum*, was found to repress not only *PPARγ*, but also *C/EBPα* and *SREBP1* expression in the 3T3-L1 cell line [48]. The lanosterol synthesis pathway, in which lanosterol is synthesized from squalene and converted into 24,25-dihydrolanosterol or water-soluble 24-methylene lanosterol, is conserved among mammals and fission yeasts [49]. An earlier study also reported that Euglena is capable of synthesizing up to 40% of the total sterols, such as squalene, triterpenes, and 4α-methylsterols, including water-soluble 24-methylene lanosterol [50]. According to these reports, it is speculated that Euglena can produce lanosterol, and thus one of the effective components in Euglena extract may be lanostane. However, the complete genome sequence of Euglena is not yet available, and whether it has the ability to convert lanosterol into lanostane remains unclear. Thus, the active component(s) in Euglena extract should be further investigated and identified to elucidate the underlying mechanism of the inhibitory effect of the extract on adipocyte-differentiation in hASCs.

## Supporting information

S1 Dataset Rawdata for 2nd revised manuscript_PLOS ONE.xlsx. (XLSX)

S1 Table Primers used in quantitative RT-qPCR for S3 Fig. (PDF)

S1 Fig Effective compound(s) in Euglena extract on adipocyte-differentiation is/are not in DMSO solvent. (A) Effect of Euglena extract by elution with DMSO (DMSO extract) on cell viability. The confluent hASCs were cultured in D/α (-) medium with DMSO (white squares) or DMSO extract (black squares) for 2 days at 37°C under 5% CO_2_ atmosphere before WST-8 assay. (B) and (C) Relative abundance of accumulated Oil Red O in hASCs treated with DMSO (B) or DMSO extract (C) compared with control (no additive, dark grey bar in (B) and (C)). Data represent mean ± SEM from 3 independent experiments. *P <0.*05* (Student’s *t*-test).
(TIF)

S2 Fig Effective compound(s) in Euglena extract could be water-soluble molecule(s). hASCs were stimulated and cultured in MDI medium to induce adipocyte-differentiation from Day 0 to Day 14 with Euglena extract (grey; 5%, dark grey; 10% or black bar; 20%) or without (as control, shown as Ctr, white bar). At Day 14, cells were fixed and stained with Oil Red O solution to determine Oil Red O content compared with control. Data represent mean ± SEM (n = 3). * *P < 0*.*05* (Student’s *t*-test).
(TIF)

S3 Fig Euglena extract represses gene expression of *aP2* and *LPL*. Relative abundance of mRNA of (left panel) *aP2* and (right panel) *LPL* in hASCs after treatment with no additive (white bar, as control) or 20% Euglena extract (black bar) for 1, 2, or 3 days compared with control. Day 0 means before induction of adipocyte-differentiation. Data represent mean ± SEM (n = 3). *P < 0.*05* (Student’s *t*-test).
(TIF)
