# Supplementary material for: Euglena extract suppresses adipocyte-differentiation in human adipose-derived stem cells
Source: PLoS One. 2018 Feb 15;13(2):e0192404. doi: 10.1371/journal.pone.0192404 (PMC5813920; doi:10.1371/journal.pone.0192404)
Supplement: S1 Table — (PDF) [file pone.0192404.s002.pdf]

S1 Table. Primers used in quantitative RT-qPCR for S3 Fig.

Primers		Sequence (direction: 5´ to 3´)
LPL	Forward	TCTTCTCGTTGGCAGGGTTG
	Reverse	CTTTCCCTTGAGGAGGAGGA
aP2	Forward	TTAGATGGGGGTGTCCTGGT
	Reverse	GGTCAACGTCCCTTGGCTTA
