# Supplementary figures and images for: Euglena extract suppresses adipocyte-differentiation in human adipose-derived stem cells
Source: PLoS One. 2018 Feb 15;13(2):e0192404. doi: 10.1371/journal.pone.0192404 (PMC5813920; doi:10.1371/journal.pone.0192404)

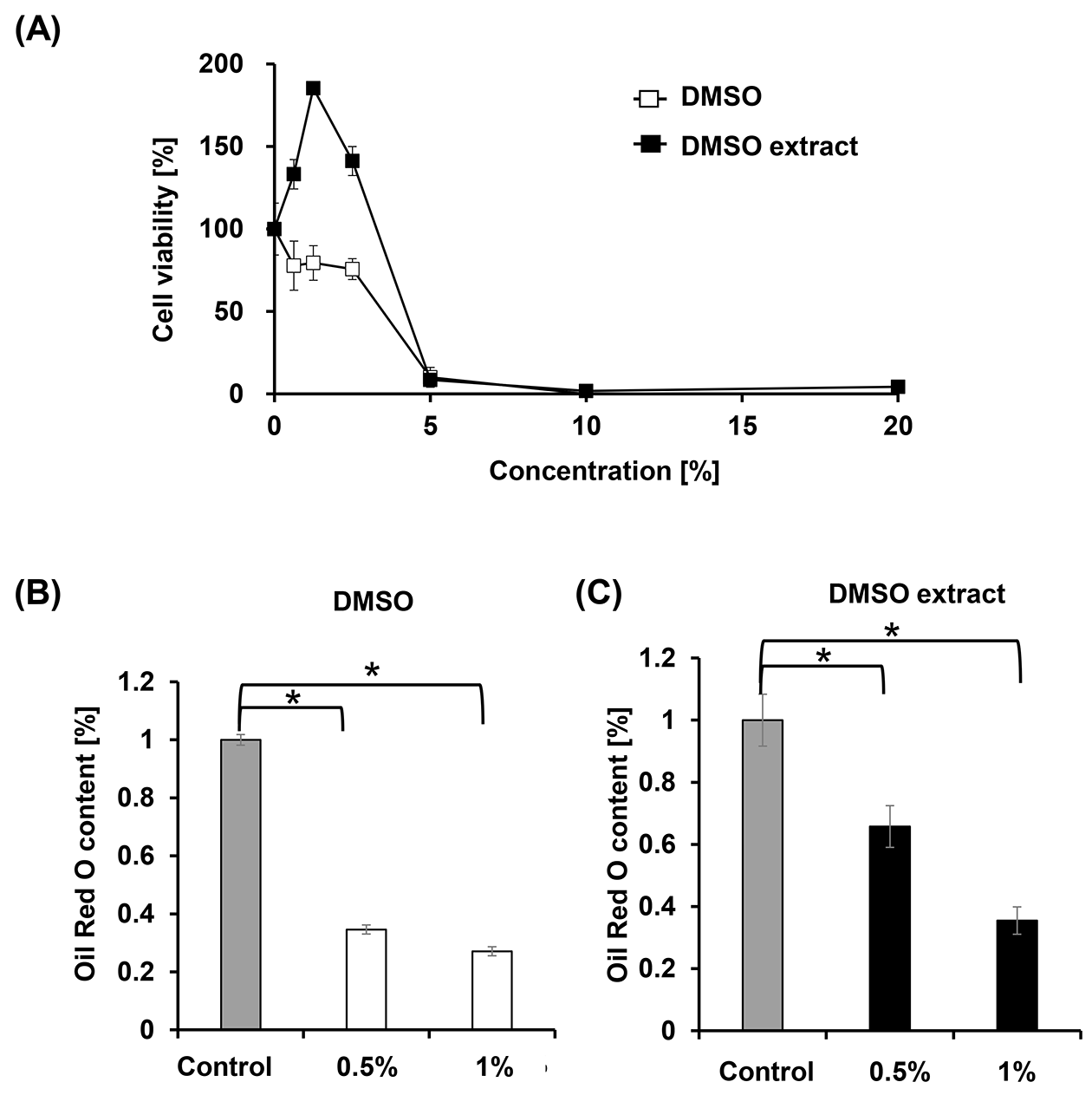

Supplement: S1 Fig — (A) Effect of Euglena extract by elution with DMSO (DMSO extract) on cell viability. The confluent hASCs were cultured in D/α (-) medium with DMSO (white squares) or DMSO extract (black squares) for 2 days at 37°C under 5% CO2 atmosphere before WST-8 assay. (B) and (C) Relative abundance of accumulated Oil Red O in hASCs treated with DMSO (B) or DMSO extract (C) compared with control (no additive, dark grey bar in (B) and (C)). Data represent mean ± SEM from 3 independent experiments. *P <0.05 (Student’s t-test). (TIF) [file pone.0192404.s003.tif]

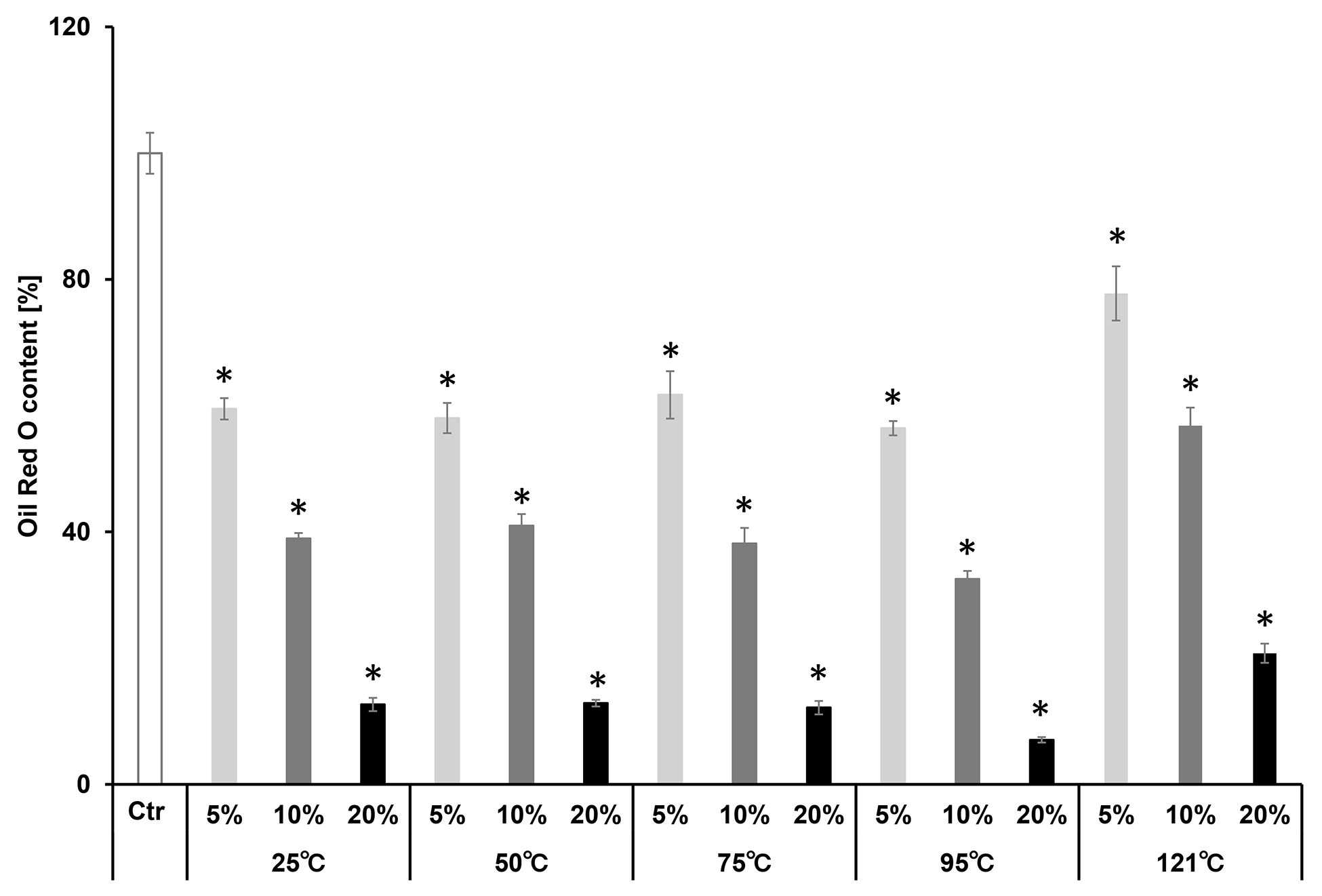

Supplement: S2 Fig — hASCs were stimulated and cultured in MDI medium to induce adipocyte-differentiation from Day 0 to Day 14 with Euglena extract (grey; 5%, dark grey; 10% or black bar; 20%) or without (as control, shown as Ctr, white bar). At Day 14, cells were fixed and stained with Oil Red O solution to determine Oil Red O content compared with control. Data represent mean ± SEM (n = 3). * P < 0.05 (Student’s t-test). (TIF) [file pone.0192404.s004.tif]

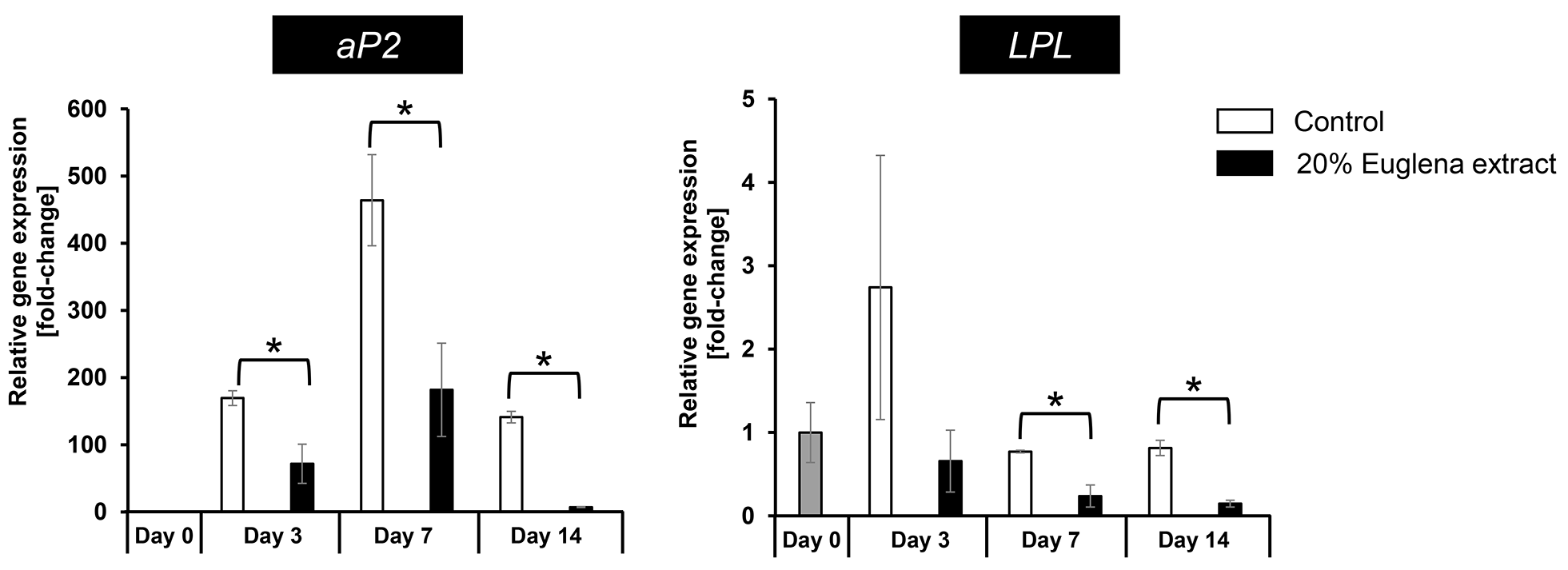

Supplement: S3 Fig — Relative abundance of mRNA of (left panel) aP2 and (right panel) LPL in hASCs after treatment with no additive (white bar, as control) or 20% Euglena extract (black bar) for 1, 2, or 3 days compared with control. Day 0 means before induction of adipocyte-differentiation. Data represent mean ± SEM (n = 3). *P < 0.05 (Student’s t-test). (TIF) [file pone.0192404.s005.tif]
